# Lessons learned from a student-driven initiative to design and implement an Organ and Tissue Donation course across Canadian medical schools

**DOI:** 10.1007/s40037-018-0454-5

**Published:** 2018-10-01

**Authors:** Alexandra Fletcher, Bing Yu Chen, David Benrimoh, Sam Shemie, Stuart Lubarsky

**Affiliations:** 1 Faculty of Medicine, McGill University, Montreal, QC Canada; 2 Department of Neurology, McMaster University, Hamilton, ON Canada; 3 Department of Psychiatry, McGill University, Montreal, QC Canada; 4 Pediatric Intensive Care Unit, McGill University Health Center, Montreal, QC Canada; 5 Neurology Unit, Montreal General Hospital, Montreal, QC Canada

**Keywords:** Undergraduate medical education, Peer-assisted learning, Course design, Competency development, Advocacy, Organ donation

## Abstract

The competencies required of the well-trained physician are constantly evolving, and medical education must adapt accordingly. In response, a growing number of influential medical education licensing and accreditation bodies have proposed frameworks that outline society’s expectations of physician competencies. In Canada, undergraduate and graduate curricula have undergone major changes to meet the specifications of the CanMEDS framework, and similar efforts are underway internationally. Nonetheless, ensuring the values enshrined within such frameworks become integral to a physician’s identity remains challenging. We believe that student-led curricular initiatives represent a novel way of approaching this shifting medical education landscape.

In this article, we reflect on lessons we learned as medical students spearheading an initiative to change how organ and tissue donation is taught in Canadian medical schools. Citing relevant medical education literature where applicable, we include a detailed description of our approach as a roadmap for students contemplating their own curricular innovations. By outlining the factors influencing this project’s implementation, as well as the benefits and limitations of student participation in curriculum reform, we offer educators a fresh perspective on optimizing the student role in this important process. Ultimately, the authors argue that not only can student participation render curricular content more accessible to learners, but that the responsibilities students take on in this role naturally lead to the development of CanMEDs-based competencies such as advocacy, scholarship, and inter-professionalism.

**Electronic supplementary material:**

The online version of this article (10.1007/s40037-018-0454-5) contains supplementary material, which is available to authorized users.

## Introduction

In recent years, medical schools are increasingly attempting to incorporate peer-assisted learning, an umbrella term for activities such as peer teaching, resource development, and curricular research [1, 2]. There is sound evidence supporting the benefits of peer-assisted learning on communication skills development, peer teacher and learner satisfaction, and resource utilization [1]. However, much of the research focuses on near-peer teaching, and pays scant attention to the role of student involvement in course development and evaluation [2].

In this article, we provide a student perspective (BYC, AF) on student-led curricular design by reflecting on our initiative to develop an Organ and Tissue Donation course for undergraduate medical education. We argue that with appropriate mentorship, not only can student participation make curricular content more learner-friendly, but the responsibilities students take on in this role naturally lead to the development of competencies common to most physician-competency frameworks [3]. Please see Tab. 1 for a summary of our reflections. Using the language of CanMEDS, we focus on advocacy, scholarship and interpersonal skills [4].

**Table 1:** A summary of lessons learned

*For medical students*
– Choose a single, well-defined topic that you are motivated to commit to. – Defining specific learning objectives, establishing a content blueprint, and developing appropriate teaching points requires a broad and preferably inter-disciplinary review process, as different stakeholders will have different and valuable perspectives. Seek advice from many sources, including both content-specific and medical education experts. This will be the beginning of your network. – Ground your work in appropriate research. Conducting a literature review will help refine the project’s aims and identify strategies to achieve them. Collecting baseline data will help define your educational focus and measure the impact of your work. a. Not only will these results influence faculty decisions regarding the validity of your work, but they are also the basis for any publications. – When collaborating with other students, as a general rule in project management, empowering those involved is more effective than directing orders. – Ensuring the long-term sustainability of your work requires high-level policy change. This is facilitated if you are able to identify motivated peers that can take over when you have to move on. Additionally, being mentored by a large organization is often associated with increased resources and opportunities to scale-up. – Endorsement adds important weight to the credibility of your work, so actively seek opportunities to showcase your work and receive funding support
*For medical educators*
– Consider how students’ topics of interest might be aligned with curricular objectives. – Encourage students to develop a scholarly, evidence-based approach to educational innovations. – Emphasize that the development of educational interventions should be informed by evidence from the medical education literature inasmuch as healthcare interventions are guided by the medical literature. – Provide literature on the curricular design process, survey development and needs assessments (Medical education-specific guides are readily available (e.g. AMEE)) – Explore with students the notion of ‘validity’ as an evidence-based ‘argument’ supporting or refuting the defensibility of an educational tool, program or intervention [13]. – Encourage students to devise strategies for ongoing course or program evaluation through the introduction of relevant frameworks, such as Kirkpatrick’s [19]. – Guide students toward relevant medical education conferences and award opportunities

## Approach

### Initiating the project

This project was conceived in 2014 during our mandate as our faculty’s public health officers. We chose to focus on organ and tissue donation in undergraduate medical education, a relatively well-defined topic that we believed would stimulate the interest of our peers. Our starting point was to contact a local organization with extensive experience in donation advocacy. Ultimately, this meeting established an ongoing partnership and represented our first link to the organ and tissue donation community.

### Defining the educational needs of the medical student population

As we wanted our recommendations for curricular change to be evidence-based, SDS, a clinician-researcher and donation advocate, helped guide our literature review of healthcare student and professional knowledge of organ and tissue donation. We then developed a needs assessment survey to measure medical student knowledge on this subject, including the ability to identify a potential donor, a competency integral to the Medical Council of Canada graduation objective 109–10: ‘[If] brain death has occurred, ensure that the appropriate donation protocol be activated.’ [5]. Students could also write down topics they believed should be taught during undergraduate medical education. In addition, a modified version served as our program evaluation tool. It was circulated following our faculty’s pilot lecture on organ and tissue donation, taught by SDS within the second year neurology block. Following the Kirkpatrick model of program evaluation, it included ‘Reaction’ and ‘Learning’ measures addressing the perceived importance of this subject in undergraduate medical education and change in knowledge scores, respectively [6].

Supervised by a member of McGill’s Centre for Medical Education (SL), we then developed a survey for all Canadian medical faculties designed to dovetail the student and faculty perspectives on organ and tissue donation education.

### Producing materials for the course proposal

Our literature review culminated in the development of a proposal for an Organ and Tissue Donation course containing a detailed list of learning objectives (see Appendix A of the online Electronic Supplementary Material). The results of our needs assessment refined the learning objectives pertaining to the donation procedure (e.g. donor identification and referral), bioethical considerations, and end-of-life communication. To ensure the proposal reflected the realities of donation, we consulted many stakeholders, including donation advocates, physicians and nurses.

The stakeholder consultation step helped validate our educational program [7]. First, by consulting widely and undertaking an iterative review process, the final product is a representative blueprint of what donation experts believe medical students should know—evidence of its *face validity* [8]. Second, and perhaps more importantly, the networking process itself built valuable grassroots support for our efforts, garnering evidence supporting its *catalytic validity*—‘the degree to which the process re-orients, focuses, and energizes participants […] in order to better transform it.’ [8].

#### Course dissemination across Canadian medical schools

To improve the visibility of our work, we sought official endorsement from our proposal’s reviewers. We then drafted position papers outlining the need for mandatory education on organ and tissue donation, which were adopted by national medical student [9] and professional [10] organizations. This flurry of activity created what John Kotter describes as ‘a sense of urgency’ [11], facilitating our project’s dissemination.

Local leadership was critical to dissemination across Canada because the process is highly faculty-dependent. Control was delegated to self-governing groups composed of students with whom we had collaborated to draft the position papers. Their early engagement in shaping the project’s vision empowered them to approach their respective faculties about incorporating elements of our proposal [8, 11]—further evidence of the project’s catalytic validity [8].

As the process was rather labour intensive, it could only succeed if students were empowered with a sense of ownership [11]. To build leadership, we teleconferenced regularly and provided research mentorship [11]. Overall, we found great inter-faculty variability regarding attitudes towards student-led initiatives (and sometimes organ and tissue donation), resulting in heterogeneous levels of progress. Currently, 11 out of 17 Canadian faculties have approved curricular changes. These figures suggest our approach has been quite successful (Kirkpatrick’s ‘Behaviour’ level) [6]. Evaluating the long-term impact on donation rates (‘Results’, the highest level of the Kirkpatrick model) requires further investigation [6].

### Laying the groundwork for sustainability

#### Individual faculty level

The results of our student surveys allowed us to mount an evidence-based argument to our faculty’s curriculum committee about the validity of our project’s aims, ensuring that the lecture gained a permanent spot within the curriculum [7]. Moreover, since our survey indicated students felt unprepared to discuss organ and tissue donation, the committee further recommended it be included within a module on ‘Delivering Bad News’ in the fourth year.

#### Organizational level

The adoption of the position papers we drafted on mandatory training in organ and tissue donation in undergraduate medical education by student organizations [9] taps into their institutional memory, mandating them to invest resources into continuing our work. On the faculty side, our survey of medical faculties, serving as both a needs assessment and a strategy development tool, helped us determine what would most help faculties improve the content of their curriculums. As a result, we are currently working on producing core competencies in organ and tissue donation for graduating medical students.

## Discussion

As exemplified by CanMEDS [4], medical schools face the task of producing graduates who are not ‘just’ medical experts. In order to foster the type of professional identity that embodies these competencies within the 4 years typically allotted to undergraduate medical education, they must be appreciated as interconnected, rather than independent ‘measurable tasks’ [12]. Our experiences have led us to believe that active involvement in curricular design is a powerful way of integrating these competencies.

### Developing new competencies

#### Advocacy

There is widespread endorsement by physician organizations for integrating advocacy into competency frameworks [13, 14], and faculties have made efforts to adjust their curricula accordingly [14, 15]. However, it would appear that this strategy needs fine-tuning—for instance, a survey of residents trained within a CanMEDS-defined curricula found the majority do not engage in advocacy [14, 16]. This has partially been attributed to how it is taught, often via didactic lectures on the social determinants of health while neglecting ‘practical activism’ skills (e.g. effective letter writing, negotiating with policymakers) [13, 15, 17].

The increasing trend to include students in advocacy teaching may indicate the tides are turning [1, 15, 18–21]. There is much to be gained: students benefit from service-learning experiences [17] which can be formalized as scholarly interactions by conducting interviews and focus groups [1]. For example, we heard eye-opening stories from donor families and recipients, and drew from these experiences by inviting them to address our class. In addition, we worked to produce ‘practice-level’ changes through the dissemination of training materials for medical students [14]. To ensure the longevity of these efforts, we engaged in ‘community and systems-level activism’ by liaising with larger organizations [14].

#### Scholarship

Authentic research experience is considered beneficial because it provides an opportunity for students to gain an appreciation for the methodologies involved, to interpret medical literature, and to foster interest in an academic career [22, 23]. Despite this, a 2015 systematic review found that although the majority of students (72%) are somewhat interested by research, among those who actually engaged, the majority did so to make their residency application more competitive [24].

In light of attitudes like these, many faculties already employ various strategies to encourage student involvement in scholarly pursuits [22–24], traditionally in fundamental or clinical research fields [23, 24]. Although we support these efforts, we believe that widening the opportunities offered has the potential to engage a greater diversity of students. Due to the dynamic and multi-faceted nature of the work, students involved in curricular design develop a broad research skillset [2, 12]. In our case, beyond content knowledge, we learned about the medical education methodologies for our needs assessment, course development and evaluation process. We also became more proficient at research-related activities, such as preparing manuscripts and presenting our work.

What kept us motivated was this project’s translational nature, which is common to advocacy-themed research [13]. As translational work requires effective stakeholder engagement, we were held accountable to a large group of people who had devoted time to helping us succeed. We also got to experience the satisfaction of witnessing our work being translated into action (first within our own milieu, then across Canada). Finally, our research experience was reinforced by an emotional dimension. We discovered that conflict can arise between research and advocacy because of their differing goals—to uncover ‘truth’ vs. to promote a cause for noble but potentially biased reasons. Although we admittedly found it challenging to temper our advocacy instinct, we ultimately embraced the responsibility of ‘wearing two hats’ once we understood the synergy between them. As neatly put by Egon Guba: ‘Relevance without rigor is no better than rigor without relevance’ [8].

#### Interpersonal skills

*Collaboration, professionalism and leadership: *Leading such a large-scale project required interactions with our many collaborators to be managed diplomatically, as any breach in professionalism could undermine an important relationship. To do this effectively without alienating or overburdening anyone meant identifying the most appropriate communication language, medium and frequency for each collaborator depending on their role [11]. The interpersonal skills we have acquired are invaluable, as literature on the benefits of peer-assisted learning [2] and service-learning [17] have shown.

### Benefits and limitations of student-led curricular design

In addition to the potential for competency development, student-led curricular design is an opportunity to improve the quality of curricular content and teaching methods, and may be interesting from a resource allocation perspective. However, important barriers must be contended with, notably lack of expertise and high rate of student turnover.

#### Impact on the curriculum

The theoretical basis of peer-assisted learning is the ‘cognitive congruence’ model, which postulates that student-designed curricula tend to be more student-centred, as students can identify a different set of needs than faculty [1, 25]. This is illustrated by the fact that in most student-led projects [1, 18–20], including our own, it was the students who perceived a gap in their curriculum. When developing course content, students usually lack content expertise and compensate by reaching out to stakeholders (*face validity *[8]), partially overcoming bias [7]. In our case, our significant stakeholder engagement efforts facilitated the project’s dissemination across Canada. To our knowledge, no other student-led project has achieved such widespread adoption. With regard to feedback, as students tend to provide more honest appraisals of peer-developed resources, this may lead to more meaningful program evaluation practices [25].

#### Resource allocation

Securing the resources necessary for student-led curricular reform, particularly the commitment of dedicated supervisors to provide longitudinal mentorship, may prove difficult [1, 2]. At the same time, however, as long as there is such a mentor, peer-assisted learning projects have consistently shown that students are capable of producing quality material that is both well received by learners and cost-effective [1, 18–20].

Curricula may take years to elaborate and must be tended to longitudinally, so student turnover is another important consideration [19]. We partially overcame this through our sustainability efforts, but despite this, the project is complex to manage and relies on our specific relationships with various individuals. It remains a challenge finding students who are able to continue our work, although the content already in place is secure. Had this project been initiated by faculty, there would likely have been better continuity [18, 20, 21].

## Conclusion

We hope that our honest presentation of what we have learned, including our missteps, is useful to the reader. To the enthusiastic medical student, we advise you find a balance between dogged advocacy and rigorous science. To the medical educator, we hope this article encourages you to take on the demanding yet rewarding task of supervising well-intentioned students and moulding them into novice medical education innovators.

## Caption Electronic Supplementary Material

The course proposal discussed in the text
