# Supplementary material for: Lessons learned from a student-driven initiative to design and implement an Organ and Tissue Donation course across Canadian medical schools
Source: Perspect Med Educ. 2018 Oct 1;7(5):332–6. doi: 10.1007/s40037-018-0454-5 (PMC6191399; doi:10.1007/s40037-018-0454-5)
Supplement: Supplementary file 1 — The course proposal discussed in the text [file 40037_2018_454_MOESM1_ESM.pdf]

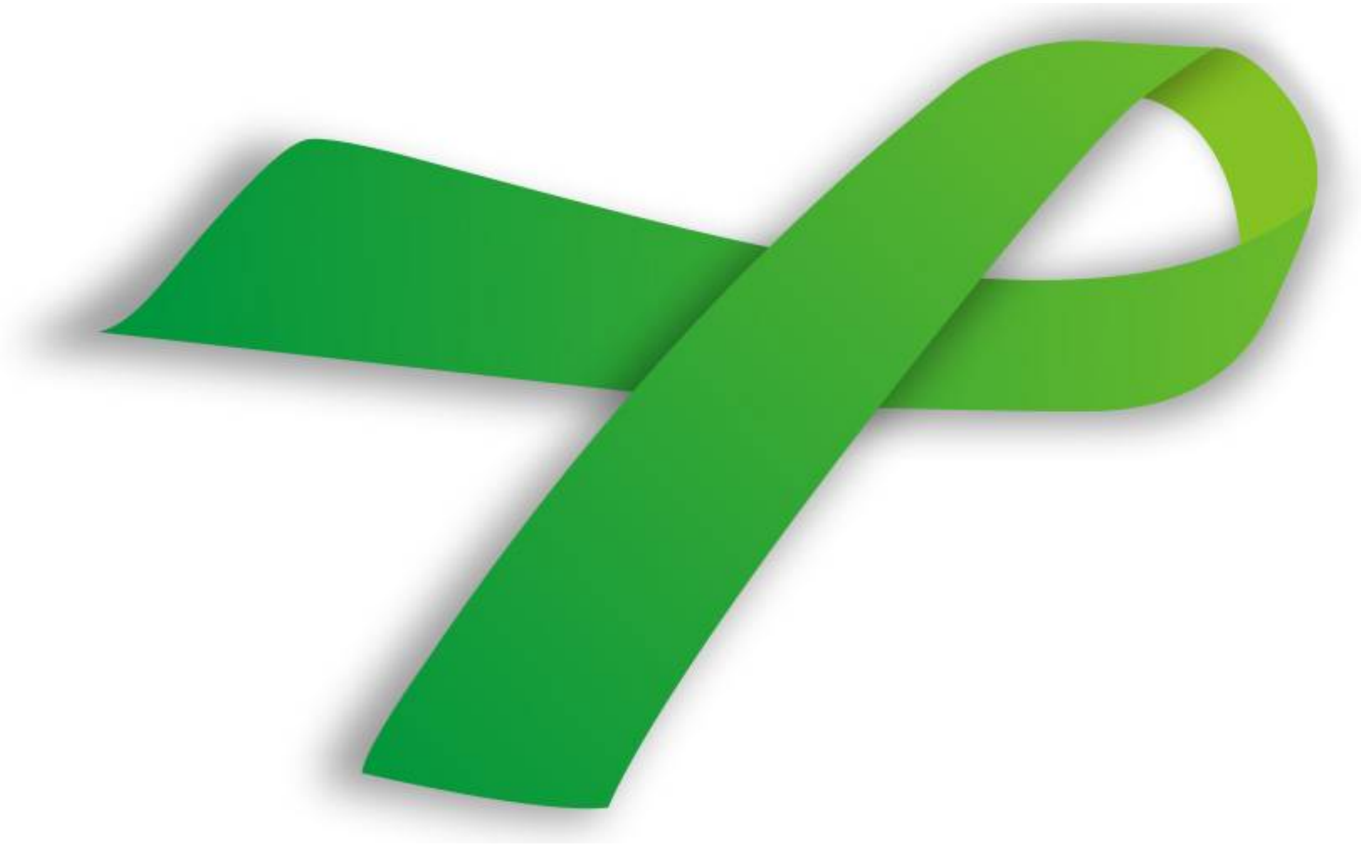

Introduction to Organ and Tissue Donation

A course for raising knowledge of and support for organ and tissue donation among future physicians in Quebec (updated September 2016)

IFMSA Québec

Canadian Blood Services
it's in you to give

Introduction to Organ and Tissue Donation

A course for raising knowledge of and support for organ and tissue donation among future physicians in Quebec (updated September 2016)

Editors: Chen, Fletcher

2016 Edition
International Federation of Medical Students' Associations – Quebec (IFMSA-Quebec)

donationmeded.com

Introduction to Organ and Tissue Donation

2016 Edition

Produced and distributed by IFMSA-Quebec

Editors: Bing Yu Chen[1,2], Alexandra Fletcher[3,4]

Co-authors: Alissa Rutman[5], Sam Shemie[6,7], Pierre Marsolais[8,9,10], Marc-André Leclair[11], Frédérick D'Aragon[12,13,14], Gilles Beaupré[15], Philippe Robert[16], Jean-François Lizé[17,18,19], Stéphan Langevin[20,21], Hugues Villeneuve[22]

Contributors: Cécile Bensimon, Christian Essman, Daniel Buchman, Jennifer Chandler, Nathalie Thiffault, Wendy Sherry

Endorsed by: Canadian Medical Association, Quebec Medical Association, Quebec Society of Intensivists, Canadian Federation of Medical Students, IFMSA-Québec, Fédération médicale étudiante du Québec, Ontario Medical Students Association, University of Toronto Medical Society, Canadian Blood Services, Transplant Québec, Héma-Québec, Mission du Dr Marsolais, Canadian National Transplant Research Program

Special thanks to Abrar Al-Jassim, Ali Damji, Aline D. Khatchikian, Allyn Walsh, Alon Coret, Anastasiya Muntyanu, Andrew Healey, Anna Oswald, Anne-Lou McNeil Gauthier, Ariane Veilleux Carpentier, Arnav Agarwal, Benjamin Cassidy, Beth-Ann Cummings, Brandon Tang, Calvin Tseng, Camille Pelletier Vernooy, Canadian Association for Medical Education, Carlos Umberto Muzlera, CBS Donation Professional Education working group, Cedrik Gignac, Christian Bourdy, Claire Touchie, Claudia Frankfurter, Claudie Dandurand Bolduc, Colin Chalk, David Alessandro Benrimoh, David Alexandre Galiano, Deanna Michelle Lammers, Denis Drouin, Denny Laporta, Donald Boudreau, Emily Hodgson, Ève-Reine Gagné, Félix Bégin, Fraser Moore, Gwynne Jones, Hélène Boisvert, Helly Goez, Jasmine Bisson, Jeff Singh, Jenna Webber, Jeremy Cepek, Jessica Luc, Jocelyn Lockyer, Johnny Huang, Josh Palay, Julie Côté-Leclerc, Julie F. Thériault, Julien Dallaire, Ken Lotherington, Laura Kim, Linda Chang Qu, Marc-André Amyot, Marie-France Giroux, Marie-Josée Hébert, Marie-Léa Guay, Martin Schreiber, Mary Tong, Mathilde Côté, Matthew Danker, Maya Deeb, McGill Centre for Medical Education, Melody Ren, Mike Moser, Mithu Sen, Nebras Warsi, Neha Arora, Normand Laberge, Rachel Ellaway, Raluca Poenaru, Reed Gillanders, Regina Taylor-Gjevre, Robert Primavesi, Samuel Bergeron, Scott Anderson, Sheena Nandalal, Sneha Raju, Stacy Tkachyk, Stéphanie Jospitre, Stuart Lubarsky, Tavis Apramian, Teresa Li, Tom Kovesi, Tracey Hillier, Valéria Akim, Victor Lam Shin Cheung, Yun Jen, Yvette Lajeunesse, and Zack Singer, for their advice and continuous support.

donationmeded.com
2016 Edition, © 2016
Introduction to Organ and Tissue Donation

All Rights Reserved

The Introduction to Organ and Tissue Donation is provided for the sole use of the recipient. No part of this publication may be used or reproduced in any form or by any means without the prior written consent permission of the publisher.

[1]Faculty of Medicine, McGill University, Correspondence via +1.514.466.2213
[2]National Officer of Public Health, IFMSA-Quebec, Correspondence via npo@ifmsa.qc.ca
[3]MSc, Faculty of Medicine, McGill University
[4]Local Officer of Public Health, IFMSA-Quebec
[5]BSc, Founder and President, McGill Students for Organ Donation Awareness
[6]MD, Pediatric Intensivist, McGill University Health Center
[7]Medical Advisor, Deceased Donation, Canadian Blood Services
[8]MD, FRCPC, Internist and Intensivist, Centre Intégré Universitaire de Santé et des Services Sociaux (CIUSSS) du nord-de-l’île-de-Montréal
[9]Medical Coordinator of Organ Procurement Centre
[10]Associate Clinical Professor, Faculty of Medicine, Université de Montréal
[11]MD, Intensivist, Centre hospitalier universitaire de Sherbrooke
[12]MD, FRCPC, MSc (epidemiology), Anesthesiologist-Intensivist, CIUSSS-CHUS
[13]Clinician Researcher, Centre de Recherche CHUS
[14]Assistant Professor, Faculty of Medicine and Health Sciences, Université de Sherbrooke
[15]CTBS, Procurement manager, Exploitation cellules souches, tissus humains et laboratoire de référence, Héma-Québec
[16]Faculty of Medicine and Health Sciences, Université de Sherbrooke
[17]Respirologist-Intensivist, CHUM

[18]Assistant Clinical Professor, Université de Montréal
[19]Assistant Medical Director, Transplant Québec
[20]Anesthesiologist-Intensivist, MD, FRCPC, Institut de Cardiologie et de Pneumologie de Québec
[21]Associate Clinical Professor in Anesthesiology, Université Laval
[22]Head of hospital development and education services, Transplant-Québec

Course Information

Title of course: Introduction to Organ and Tissue Donation

Program: Medicine – M.D. & M.D., C.M.

Term in which it will be offered: Preclerkship or clerkship – preferably in a neurology unit

Total hours: 6 hours
- 1.5 hour of theory-based classroom lecture on organ donation – meeting objectives for the qualifying examination of the Medical Council of Canada (MCC)
- 3 hours of small group learning on organ donation – meeting objectives for the qualifying examination of the Medical Council of Canada (MCC)
- 0.5 hour of theory-based classroom lecture on tissue donation – recommended
- 1 hour of theory-based classroom lecture on bioethics in organ and tissue donation – recommended

Prerequisite: Previous teaching on neurological determination of death is recommended.

Course Description: This introductory course on organ and tissue donation is based on classroom lectures and small group learning, and aims at raising knowledge of and support for donation among future physicians. Topics covered include context of organ and tissue donation, organ and tissue donation procedure – identification of potential organ and tissue donor, referral to organ and tissue procurement centres and communication with families – and bioethical issues in organ and tissue donation.

Rationale

Through transplantation, organ and tissue donation is responsible for saving thousands of lives every year worldwide. “In all countries in which deceased organ donation has been initiated, the therapeutic potential of deceased organ donation and transplantation should be maximized.” (9) Unfortunately, a discrepancy between the demand for organs and tissues and the availability of donors exists, in Canada and internationally. Success in organ and tissue donation relies highly on the knowledge of health care professionals. (1) Given this fundamental role physicians play in the identification and referral of potential donors, it comes as no surprise that the evidence supports enhanced undergraduate medical education as a powerful method to improve organ and tissue donation rates. (2) Despite this overwhelming evidence, a gap still exists in the way that organ and tissue donation is currently being taught in the medical curriculum. (3) Our objective is to implement an evidence-based, expert-supported course on organ and tissue donation into the Quebec medical curricula in order to raise knowledge of and support for donation among future physicians. If this course is adopted, we expect to ultimately improve organ donation rates in the province and we expect to quantify this change.

In addition to the inherent importance of organ donation, the topic is also part of the Medical Council of Canada (MCC) objectives, an additional reason to include it at the undergraduate medical level.

The present introductory course described in this proposal, available during the preclinical years in the forms of theory-based classroom lectures, cultivates medical students' interests early in their education in the absence of barriers to learning such as lack of time and false sense of competency and encourages them to pursue a profession in donation and transplantation or a related field. (2) We believe that the implementation of this course will be very feasible and will create a unique educational experience for medical students, who will be key players in shaping the organ and tissue donation system of tomorrow.

MCC Objectives (4)[1]

Head Trauma / Brain Death / Transplant Donations

Rationale
While most head trauma is mild and not associated with long-term sequelae, clinical examination may fail to detect serious intracranial injuries that are evident on radiological imaging. Therefore, it is imperative to recognize head injured patients that require additional diagnostic imaging. When brain death has occurred, organ transplantation should be considered.

Causal Conditions
1. Skull fracture, penetrating injury
2. Hemorrhage, hematoma (subdural, epidural, subarachnoid, shaken baby syndrome)
3. Cerebral contusion
4. Edema (midline shift)

Key Objectives
Given a patient with a head/brain injury, the candidate will diagnose the cause, severity and complications. In particular, the candidate will, based on the mechanism of injury and the clinical findings, determine the appropriate management plan and select appropriate imaging and ongoing surveillance. In cases where brain death has occurred, *discuss organ donation with the next of kin*.

Enabling Objectives
Given a patient with a head/brain injury, the candidate will
1. list and interpret critical clinical findings, including those derived from
    a. a history aimed at determining if the head injury was severe, or associated with complication (e.g., mechanism of injury, loss of consciousness);
    b. a physical examination aimed at determining if the head injury was severe, or associated with complication (e.g., ecchymosis behind ear);

[1] Original text from MCC

        c. a repeat history or examination aimed at detecting evolving pathology;
        d. clinical signs of brain death;
2. list and interpret critical investigations, including
    a. determination as to whether the patient requires urgent brain imaging;
    b. confirmation of brain death with appropriate investigations;
3. conduct an effective initial management plan, including
    a. determine if the patient requires specialized or urgent care;
    b. *in a patient whose head injury has caused brain death, but whose heart is still beating, communicate this information to the transplantation team (or equivalent) if the deceased patient or the family have indicated a desire to donate organ(s);*
    c. *if there is no indication that organ donation has been considered, counsel the family about the possibility.*

Cardiac Arrest

Rationale
Cardiac arrest is life threatening and relatively common, particularly in the hospital setting. Timely basic and advanced cardiac life support improves patient survival.

Causal Conditions
1. Coronary artery disease
2. Cardiac conduction abnormalities
3. Myocardial abnormalities
4. Non-cardiac causes (e.g., pulmonary embolus)

Key Objectives
Given a patient who presents with cardiac arrest, the candidate will be able to initiate immediate acute cardiac life support, and construct an appropriate subsequent management plan.

Enabling Objectives
Given the patient with cardiac arrest, the candidate will
1. list and interpret critical clinical findings, including
    a. pulseless circulatory state;
    b. features that may help determine the cause of the arrest;
2. list and interpret critical investigations
3. construct an effective management plan, including
    a. initiation of basic and acute cardiac life support protocols;
    b. communication with family members concerning the event, including
        i. outcome
        ii. breaking bad news
        iii. organ donation
        iv. autopsy request.

Learning Objectives

This course will help medical students to
- foster an interest in and awareness of organ and tissue donation;
- be acquainted with the context of organ and tissue donation;
- have a general understanding of the organ and tissue donation procedure;
- understand major bioethical issues in organ and tissue donation.

Course Outline

Teaching items in italics are optional. These items are considered supplemental materials.

Lecture 1 – Designed to meet MCC Objectives

Title: Organ Donation

Duration: 1.5 hour

Instructor: Intensivist, organ donation resource nurse, representative from organ procurement organisation

Learning objectives:
- Understand the basic principles of organ donation
- Appreciate the benefits of organ and tissue donation and transplantation
- Be acquainted with the key organ donation statistics
- Know the characteristics for identifying organ donors
- Understand donation registries and referral of potential organ donors
- Appreciate families' attitudes, fundamental strategies for obtaining donation consent and barriers to family approach
- Understand the transplant waiting list
- Acknowledge the barriers to donation availability and physicians' responsibilities

Topics covered:

Context of organ donation
- Basic principles of organ donation
    - Duty to serve the needs of potential transplant recipients in an ethical, legal, safe and equitable manner
    - Providing the opportunity to donate and to respect the donor's wishes without compromising the duty of care to the dying patient or living donor
    - Two types of donors: deceased and living donors

- *History of neurological determination of death and organ and tissue donation and future perspectives*
- *Patient stories*
- Benefits of organ and tissue donation and transplantation
    - Recipient: Transplantable organs and tissues, patient survival rates according to transplanted organs, number of patients benefited from donation
    - Donor's family: Overcoming grief over loss of loved one
    - Society: Cost-benefit analysis of kidney transplantation compared to dialysis
- Organ donation statistics
    - Number of organ donors, transplanted patients, patients on the waiting list, deceased patients on the waiting list, lifetime probability of receiving a transplant
    - Statistics in Canadian provinces and other countries

Organ donation procedure
- Overview of the organ donation procedure
    - Produced by Transplant Québec, Association des Conseils de médecins, dentistes et pharmaciens and Association québécoise d'établissements de santé et de services sociaux
    - Used in all healthcare institutions
- Step 1: Identification
    - Breakdown of the number of hospital deaths, potential donors and actual donors
        - Emphasis on the rarity of donors and importance of identifying all potential donors
        - Provincial law on mandatory potential donor referral
            - In Quebec, article 204.1 of LSSSS: Loi facilitant les dons d'organes et de tissus
    - Causes of death in organ donors and course of disease leading to poor prognosis
    - Basic criteria for potential organ donor
        - Imminent or established death in hospital, severe brain injury, mechanical ventilation
        - Identification in a context of end-of-life care, usually in the emergency department, intensive care unit and critical care unit
    - Contraindications to organ donation
        - Always contact organ procurement organisation at this stage to ensure that no donor is missed
        - Consent status in the registries is verified at this stage for convenience only, the organ procurement organisation does not intervene before diagnosis is made
    - Neurological determination of death (NDD)
        - Concept, criterias, physical examination, ancillary tests (angiography, scintigraphy)
    - Donation after cardiocirculatory death (DCD)
        - Concept
    - Diagnosis of death completed by 2 physicians
    - Common misconceptions about potential organ donors
        - Age, religion, funeral plans, quality of care for consented patients

    - Summary potential organ donor identification flowchart
- Step 2: Referral
    - *Roles of organ donation resource nurse and organ procurement organisation in the organ donation procedure*
    - Understanding expression of consent through donation registries, for minors and adults
        - Articles 43 and 44 of Code civil du Québec
        - Percentage of population in favour of organ donation
        - Importance of communicating wishes with next of kin
    - Referral and verification of patient's consent status in the registries
        - Stats: Higher referral rate results in higher donation rate
- Step 3: Communication of diagnosis / prognosis and presentation of the option of organ donation
    - Families' attitudes
        - Donation provides meaning to the loss of their loved one and allows families to honour wishes
            - Importance of offering the option of donation
    - Procedure and strategies
        - The treating physician announces and explains the poor and irreversible prognosis
            - Provide a quiet and private space
            - Use a family-centered approach and provide continuous support to families
            - Ensure that the family understands the prognosis
                - Use of visual aids (i.e. brain scan)
            - Reassurance in optimal care provided by healthcare team
        - Discuss withdrawing life-sustaining therapies in the case of DCD
        - Present the option of organ and tissue donation as an option in end-of-life care
            - Right time, right person, right method
                - Right time
                    - Once the family understands that the patient will not survive
                    - When the family initiates the discussion about organ donation
                    - When the decision is made to withdraw life-sustaining treatment
                - Right person
                    - Team-based family approach
                    - The donation option is presented by the treatment team member (physician, nurse, spiritual advisor, social worker) who has the best relationship with the patient's family or the most experience and skill in communicating with families
                - Right method
                    - Decoupling
            - Project positivity about obtaining consent from families
            - Enquire about the reason for not donating
    - Barriers to family approach

        - Denial of poor prognosis in a context of shock and difficult circumstances
        - Misunderstanding of the concept of NDD or of the context of end-of-life care
        - Lack of time resource
- Step 4: Evaluation, eligibility and procurement
    - *Brief presentation of the following steps*
        - *Donor management*
        - *Donor eligibility evaluation – medical history and investigations (e.g. biopsy)*
        - *Organ procurement*
    - Brief presentation of the transplantation waiting list
        - Stats: Average waiting time per organ
    - *Donor's family follow-up and bereavement support*
        - *Examples in Quebec*
            - *Post-donation follow-up by Transplant Quebec*
            - *Donor Recognition Ceremony*
            - *CODA Recognition Ceremony for Families of Deceased Donors*
- Conclusion
    - Challenges to donation availability
        - Community
            - Ethnocultural (ethnic and cultural minorities are less likely to donate), religious (e.g. some Jewish communities object to NDD), personal culture, distrust, fear, apathy
        - Healthcare system
            - Identification of potential donors (all healthcare workers in ED, ICU and CCU should contribute towards identification, especially respiratory therapists), family approach, donor management, hospital resources (access to intensive care unit and operating room) and engagement
    - Physician's responsibilities
        - Identification, referral and, when appropriate, presentation of the option of organ donation
        - Awareness and advocacy

Small Group Learning 1 – Designed to meet MCC Objectives

Title: Organ Donation

Duration: 3 hours

Instructors: Intensivist, organ donation resource nurse, representative from organ procurement organisation

Learning objectives:
- Know the characteristics for identifying organ donors, including neurological determination of death (NDD) and donation after cardiac death (DCD)
- Understand donation referral of potential organ donors

- Appreciate families' attitudes, fundamental strategies for obtaining donation consent and barriers to family approach
- Understand an overview of the procedure for procurement
- Acknowledge the barriers to donation availability and physicians' responsibilities

Case summary: An unconscious 37-year-old woman suffered from severe intracranial hemorrhage following a motor vehicle accident and is in critical condition in ICU. Over the past two days, she is surrounded by her family, who is visibly worried about her prognosis. Although the patient is ventilated and that her heart is beating, brain death is suspected based on neurological exam.

Guiding questions:

- What is the clinical definition of brain death? Describe the ancillary tests used to confirm the diagnosis of brain death.
- What are the physiological mechanisms of brain death? What are the clinical scenarios that most commonly lead to brain death (and organ donation)?
- What are the clinical criteria to be considered a potential organ donor? What additional information will Transplant Quebec verify about the patient?
- Once NDD has been established, how will you approach the family, and how will you describe brain death to them?
- How do you bring up the topic of donation? How would you respond to hesitations from the family? What should you do in the case of a refusal?
- Once the patient has been consented for organ donation, what is the protocol between consent and the operating room?
- Describe your understanding of DCD. How does the procedure of DCD differ from NDD?
- What do you consider to be the main barriers to improving organ donation rates? Discuss potential strategies to overcome these barriers.

Lecture 2 – Recommended

Title: Tissue Donation

Duration: 0.5 hour

Instructor: Representative from tissue procurement organisation

Learning objectives:
- Be acquainted with the key tissue donation statistics
- Know the characteristics for identifying tissue donors
- Understand referral of potential tissue donors
- Appreciate the differences and similarities in family approach between organ and tissue donation

Topics covered:

Context of tissue donation

- Tissue donation statistics
  - Number of tissue donors and transplanted patients
  - Projected needs

Tissue donation procedure

- Overview of the tissue donation procedure
- Step 1: Identification
  - Percentage of deceased patients in hospital eligible for tissue donation
  - Criteria for potential tissue donor
  - Causes of death in tissue donors
  - Common misconceptions about potential tissue donors – autopsies, coroner's case
  - Contraindications to tissue donation
  - Summary potential tissue donor identification flowchart
  - Roles of tissue procurement organisation in the tissue donation procedure
- Step 2: Referral
  - Verification of patient's inscription in the registries
- Step 3: Presentation of the option of tissue donation
  - Similarities and differences with organ donation
- *Step 4: Evaluation and management*
  - *Donor eligibility evaluation – medical history*
  - *Donor management*
- *Step 5: Procurement by tissue procurement organisation*
  - *Recovery of tissues*
  - *Bank of tissues – objective tests and processing techniques*
- Summary of similarities and differences with organ donation

Lecture 3 – Recommended

Title: Bioethical issues in Organ and Tissue Donation

Duration: 1 hour

Instructors: Bioethicist

Learning objectives:

- Be acquainted with key laws and regulations on organ and tissue donation
- Appreciate ethical issues surrounding definition of death, organs and tissues procurement and allocation
- Understand current controversial topics in organ and tissue donation

Topics covered (8):

- Brief introduction to bioethics
- Brief overview of organ and tissue donation law and regulations in Canada
    - Quebec
        - Bill n°125 : An Act to facilitate organ and tissue donation
        - Loi sur les services de santé et les services sociaux
    - Accreditation Canada
- Ethical issues in defining death
    - Social, historical, cultural, religious, and spiritual considerations
    - Neurological Determination of Death (NDD)
    - Organ donation after circulatory death (DCD)
- Retrieving organs and tissues
    - Duties and obligations towards patients and conflicts of interest
        - The Dead Donor Rule
        - Separate medical teams
        - Donation after physician-assisted suicide
        - Pre-death care in DCD patients (e.g. heparin, antibiotics and corticosteroid administration, bronchoscopy) may not be in their best interests
    - Family veto to donor's expressed wishes
    - Presumed consent
- Allocating Organs and Tissues
    - Ethical principles in allocation
    - Criteria for recipient selection
        - Psychosocial considerations
        - Re-transplants
        - Multi-organ transplants
        - Age
        - Non-residents and uninsured patients
    - Organ donation systems
        - UK Transplant
        - Israel's priority system (2008)
- Controversial topics in organ and tissue donation
    - Transplant tourism
        - Declaration of Istanbul (2008)
    - Financial incentives for living donation
    - Public solicitations

Alternative teaching methods

Small group teaching sessions have gained popularity in medical education as they encourage self-directed learning, increase student interest and enhance retention of knowledge and skills (7). Several general themes presented in the lectures may be suitable for the small group teaching format, either as a replacement of the lecture or as a reinforcement of the content taught during the lecture. Such themes include but are not limited to: organ and tissue donation procedures, communication with donor's family and bioethical issues in organ and tissue donation.

Other teaching methods, such as mandatory online modules and simulation-based learning, could also replace conventional didactic lecture teaching format to address the learning objectives above.

Student Evaluation

Knowledge acquired during lectures will be evaluated during written examinations according to faculty regulations.

Course Evaluation

Students are encouraged to provide feedbacks and suggestions to this course through formal surveys, and data collected will be used only for research purposes.

Required Text

PowerPoint and PDF materials are provided to students.

Teaching Resources

Lecturers may be appointed by the faculty itself. Recommended areas of expertise can be found in the Course Outline section.

Impact on the Medical Curriculum

The content of this course complements well with the neurology unit during pre-clinical years, especially following the lecture on neurological determination of death. There should not be any conflicts with other courses.

Course Implementation and Future Prospective

This course should be implemented prior to July 2018. As new research is being done and best practices are being shared among participating faculties, revisions to this course will be expected.

References

1. Irving MJ, Tong A, Jan S, Cass A, Chadban S, Allen RD, et al. Community attitudes to deceased organ donation: a focus group study. Transplantation. 2012;93(10):1064-9.
2. Essman, C. C. and D. J. Lebovitz (2005). "Donation education for medical students: enhancing the link between physicians and procurement professionals." Prog Transplant **15**(2):124-128.
3. Nelson EW, Mone MC, Nelson ET, Hansen HJ, Gawlick U, Alder S. Evolution of trends in the live kidney transplant donor-recipient relationship. Transplant Proc. 2013;45(1):57-64.
4. The Medical Council of Canada & Le Conseil médical du Canada – Objectives For The Qualifying Examination
5. Standard organ donation procedure, Transplant Quebec
6. Cadre organisationnel en don d'organes et en don de tissus, Transplant Quebec
7. Meo SA. Basic steps in establishing effective small group teaching sessions in medical schools. Pak J Med Sci. 2013 Jul;29(4):1071-6. Review.
8. Veatch, R. (2002). Transplantation Ethics. Washington: Georgetown University Press.
9. The Declaration of Istanbul on Organ Trafficking and Transplant Tourism
